# Aortic root parameter analysis for transcatheter aortic valve implantation in the Pakistani population: a retrospective study

**DOI:** 10.1186/s43044-024-00569-0

**Published:** 2024-10-19

**Authors:** Muhammad Suleman, Maryam Masoud, Muhammad Ishaq Khan, Ihsan Ullah, Abid Ullah, Rafi Ullah Jan, Shah Zeb, Umar Ashfaq, Ali Raza, Mohammad Waleed

**Affiliations:** Peshawar Institute of Cardiology, Peshawar, Pakistan

**Keywords:** TAVI, Aortic annulus size, CT valve sizing, Valve selection

## Abstract

**Background:**

Transcatheter aortic valve implantation (TAVI) is a growing treatment for aortic stenosis, but anatomical variations exist across populations. Asians tend to have smaller aortic annuli and higher bicuspid valve morphology compared to Westerners, potentially impacting TAVI valve selection and outcomes. This study analyzes aortic root parameters in Pakistani patients undergoing TAVI.

**Results:**

We conducted a retrospective analysis of 78 patients who underwent TAVI at the Peshawar Institute of Cardiology from January 2021 to March 2024. Pre-procedural CT scans were analyzed for aortic annulus diameters, area, and other relevant parameters. The mean age was 72.41 years (SD ± 11.99), and 61.5% were male. Aortic annulus diameters (minimum, mean, maximum) were 21.18 mm (SD ± 3.99), 24.21 mm (SD ± 3.93), and 27.49 mm (SD ± 4.43), respectively. Bicuspid aortic valves were present in 34.61% of patients.

**Conclusions:**

Our findings suggest that Pakistani patients undergoing TAVI may have aortic root dimensions comparable to Western populations, with a substantial prevalence of bicuspid valves. However, coronary heights were similar to those reported in Asian populations with smaller annuli. Further studies are needed to assess TAVI outcomes in Pakistani patients and determine if tailored valve sizing strategies are required.

## Background

Transcatheter aortic valve implantation (TAVI) has emerged as a life-saving treatment for patients with severe aortic stenosis, offering a minimally invasive alternative to surgical aortic valve replacement (SAVR) [1, 2]. Accurate sizing of the prosthetic valve is crucial for the success of TAVI procedures, and the aortic annulus diameter serves as a key parameter in this process [3]. Current guidelines emphasize the importance of pre-procedural computed tomography (CT) for meticulous measurement of the aortic annulus to ensure optimal valve selection and minimize the risk of complications [4, 5].

However, anatomical variations exist across different populations [6]. Studies have shown that patients of Asian descent tend to have smaller aortic annulus diameters compared to their Western counterparts [7]. The frequency of bicuspid aortic valve is also higher in Asian population [8]. This highlights the potential need for tailored sizing strategies and reference ranges when performing TAVI in Asian populations.

Given the growing adoption of TAVI in Pakistan, it is essential to understand the specific aortic root parameters characteristic of this population. This study aims to analyze the aortic root parameters measured through pre-procedural CT scans in a cohort of Pakistani patients undergoing TAVI. By establishing reference ranges for this specific population, we can contribute to more accurate valve sizing and potentially improve TAVI outcomes in Pakistan.

## Methods

### Study design and setting

This retrospective cross-sectional study was conducted at the Peshawar Institute of Cardiology. Data were collected from January 2021 to March 2024.

### Sample selection

Patients of both genders who underwent computed tomography (CT) with the transcatheter aortic valve implantation (TAVI) protocol were included. Only those deemed suitable candidates for the Evolut R TAVI valve based on their anatomical parameters were selected. The exclusion criteria were as follows:

1. Presence of severe motion artifacts on CT images.
2. Undergoing CT for pure aortic regurgitation.
3. Diagnosis of congenital aortic stenosis.

### CT acquisition

All patients underwent multi-detector computed tomography (MDCT) using a Canon Aquilion Prime SP 80-slice CT scanner with a 3-phase vHP dedicated TAVI protocol. One patient had MDCT performed at an external hospital using a Philips machine (model unknown). The MDCT protocol involved the following:

- Patients were positioned supine with arms above the head.
- A timing bolus with the region of interest in the ascending aorta was used.
- The slice thickness was set to 0.5 mm with a 512 × 512 matrix size.
- A cranio-caudal acquisition was taken during a breath-hold, followed by a non-gated CT to the carina, a gated CT to the inferior margin of the heart, and a non-gated CT scan extending to the upper thighs.
- 100 ml of non-ionic tri-phasic contrast was used during acquisition.
- Both systolic (R–R 30–35%) and diastolic (R–R 85%) phases were reconstructed for all patients.

### Data collection and analysis

After obtaining ethical review committee approval, retrospective data were collected on 78 patients who met the inclusion and exclusion criteria. All these patients underwent CT TAVI analysis using Vitrea Extend (Canon Medical System Corp.). Data were extracted from pre-procedural TAVI reports and procedural records, focusing on aortic annulus measurements (minimum and maximum diameters), derived diameters, annular area, sinus width, sinus heights, coronary heights, and valve type (bicuspid, tricuspid).

Descriptive statistics were used to summarize quantitative data as mean ± standard deviation (SD) and qualitative data as frequencies and percentages. Stratification was employed to control for potential confounders such as age, gender, weight, height, and comorbidities. Post-stratification chi-square tests were applied, with a significance level set at ≤ 0.05. Statistical analyses were performed using SPSS version 22 and R software.

## Results

We analyzed the CT scans of 78 consecutive patients scheduled for TAVI at our institute between January 2021 and March 2024. Of these patients, 48 (61.5%) were male, and 30 (38.5%) were female, with a mean body surface area of 1.81 ± 0.16 m^2^. The baseline characteristics of the study population are summarized in Table 1.

**Table 1:** Baseline characteristics

		Count (Percentage)	Mean ± SD
Gender	Male	48 (61.5)	
	Female	30 (38.5	
Age			72.41 ± 11.99 Years
Dyspnea NYHA class	NYHA Class I	0 (0.0)	
	NYHA Class II	45 (57.7)	
	NYHA Class III	28 (35.9)	
	NYHA Class IV	5 (6.4)	
Syncope	No	62 (79.5)	
	Yes	16 (20.5)	
Height			1.63 ± 0.08 cm
Weight			73 ± 11.76 kg
Body surface area			1.81 ± 0.16 m^2^
Basal metabolic index			27.66 ± 5.27 kg/m^2^

Table 2 presents the echocardiographic characteristics of the patients. The mean ejection fraction (EF) was 42.5%, with average mean and peak gradients within the severe aortic stenosis range.

**Table 2:** Echocardiographic descriptive statistics

	N	Minimum	Maximum	Mean	Std. deviation
Ejection fraction	78	25.00	60.00	42.5000	11.44581
EOA	78	.70	1.09	.8953	.11550
MPG	78	41	73	56.48	10.517
PPG	78	61	110	84.72	15.776

**Fig. 1:**
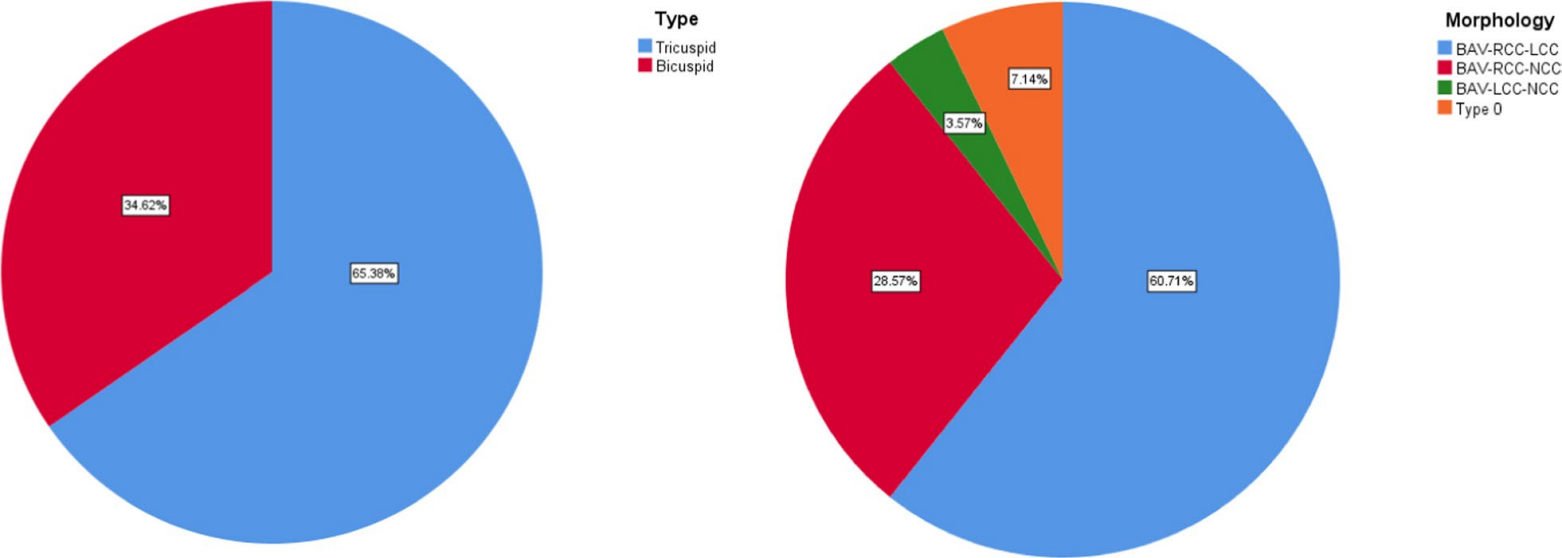
Morphology of aortic valve. *BAV* Bicuspid valve, *RCC* Right coronary Cusp, *LCC* Left coronary Cusp, *NCC* Non-coronary cusp

Figure 1 illustrates the aortic valve morphology as determined by CT scans. One in three patients exhibited bicuspid valve morphology, with right and left cusp fusion being the most common type.

Table 3 provides a detailed analysis of CT scan parameters for the patient population. The mean annular diameter was 24.21 ± 3.93 mm. There was no statistically significant difference between tricuspid and bicuspid morphology across all parameters.

**Table 3:** CT scan parameters

Parameter	Total *n* = 78	Tricuspid *n* = 51 (65.38%)	Bicuspid *n* = 27 (34.61%)	*P*-value
Annulus diameter min	21.18 (± 3.99)	21.39 (± 3.72)	20.78 (± 4.51)	0.35
Annulus diameter max	27.49 (± 4.43)	28.00 (± 3.78)	26.53 (± 5.41)	0.43
Annulus diameter mean	24.21 (± 3.93)	24.49 (± 3.37)	23.68 (± 4.85)	0.22
Annulus perimeter	76.92 (± 12.34)	77.99 (± 10.96)	74.90 (± 14.60)	0.27
Annulus perimeter derived diameter	24.48 (± 4.00)	24.81 (± 3.50)	23.84 (± 4.81)	0.50
Annulus area	466.93 (± 151.22)	479.08 (± 139.70)	443.99 (± 171.33)	0.27
Annulus area derived diameter	24.04 (± 3.89)	24.41 (± 3.45)	23.33 (± 4.61)	0.35
LVOT diameter min	20.33 (± 4.16)	20.43 (± 3.92)	20.15 (± 4.63)	0.16
LVOT diameter max	28.81 (± 4.66)	29.29 (± 4.19)	27.91 (± 5.41)	0.53
LVOT diameter mean	24.57 (± 4.23)	24.84 (± 4.01)	24.07 (± 4.65)	0.31
LVOT perimeter	33.91 (± 4.60)	33.40 (± 4.31)	34.86 (± 5.03)	0.23
STJ minimum diameter	28.58 (± 3.98)	28.32 (± 3.86)	29.08 (± 4.22)	0.11
STJ maximum diameter	30.14 (± 4.43)	29.87 (± 4.43)	30.66 (± 4.48)	0.35
SOV Left cusp diameter	32.43 (± 4.01)	32.56 (± 4.00)	32.17 (± 4.10)	0.21
SOV Right cusp diameter	30.73 (± 3.86)	30.84 (± 3.81)	30.50 (± 4.02)	0.24
SOV Non-cusp diameter	32.40 (± 4.04)	32.55 (± 3.88)	32.11 (± 4.40)	0.42
SOV height left cusp	21.77 (± 3.50)	21.82 (± 3.79)	21.68 (± 2.98)	0.35
SOV height right cusp	21.64 (± 3.51)	22.10 (± 3.63)	20.74 (± 3.16)	0.54
SOV height non-cusp	20.96 (± 3.78)	21.31 (± 3.92)	20.27 (± 3.47)	0.34
Left coronary height	13.57 (± 2.66)	13.63 (± 2.91)	13.48 (± 2.15)	0.32
Right coronary height	15.79 (± 2.82)	16.23 (± 2.86)	14.95 (± 2.60)	0.34
Left femoral diameter	6.37 (± 1.63)	6.1 (± 1.48)	6.64 (± 0.23)	0.82
Right femoral diameter	6.56 (± 1.09)	6.9 (± 1.52)	6.22 (± 0.73)	0.75
Left internal iliac artery	6.52 (± 1.03)	6.2 (± 0.96)	6.84 (± 0.25)	0.96
Right internal iliac artery	7.11 (± 1.77)	6.9 (± 1.88)	7.32 (± 0.91)	0.63
Left common iliac artery	7.30 (± 1.77)	7.1 (± 0.41)	7.5 (± 0.11)	0.80
Right common iliac artery	8.0 (± 2.04)	7.8 (± 1.79)	8.2 (± 1.64)	0.94

Table 4 shows that 87% of patients were deemed suitable for the Evolut R device, while 13% were rejected. The most common reason for rejection was an annulus size larger than permitted by the device’s instructions for use.

**Table 4:** Valve suitability

	Count (%)
*Suitable*	
Accepted	68 (87.1)
Rejected	10 (12.9)
*Rejection reason*	
Annulus is too small	2 (2.6)
Annulus is too big	8 ( 10.3)

## Discussion

Our study revealed significant findings, including the mean minimum and maximum aortic annular diameters, which were 21.31 ± 3.96 mm and 27.59 ± 4.45 mm, respectively. The mean annular perimeter was 77.25 ± 12.32 mm, and the mean coronary heights were 13.64 ± 2.65 mm (left) and 15.87 ± 2.82 mm (right). Additionally, 27 patients (34.61%) were found to have a bicuspid aortic valve morphology, highlighting the prevalence of this condition in our cohort.

In recent years, TAVI has gained widespread acceptance as a viable treatment option for severe aortic stenosis, particularly in Western populations. However, its adoption in Asian countries has been relatively slow. Encouragingly, there has been a notable increase in the integration of TAVI into clinical practice across Asia, reflecting a shift toward broader acceptance of this minimally invasive procedure in the region.

Among many prerequisites required for pre-procedural planning of TAVI, accurate sizing of the valve derived from CT measurements is of paramount importance. It has been shown that TEE-based aortic root measurements have been resulted in significant rate of paravalvular leak and other procedure related complications owing to incorrect measurements and CT derived aortic perimeter has been the most sensitive factor for transcatheter heart valve sizing [9].

Unfortunately, till date there is limited representation of Asian population in various TAVI trials. Previous studies have shown that Asian populations have got smaller aortic roots as compared to Western population. Small aortic annulus has its own inherent risk of complications such as aortic annulus rupture with excessive sizing, patient prosthesis mismatch, and coronary artery occlusion [10]. So, Asian population can be considered more to be at risk of these serious complication than their Western counterparts. This implication emphasizes more on the development of transcatheter heart valves with different sizes and profile tailored to the anatomical characteristics of Asian population.

There is one Japanese study comparing Japanese and European population for their aortic annular sizes. The study has shown significantly smaller minimum and maximum aortic annular diameters in Japanese population than European population (19.4 ± 2.0 mm vs. 22.6 ± 2.3 mm, *p* < 0.01; 24.7 ± 1.9 mm vs. 27.6 ± 2.5 mm, *p* < 0.01, respectively) along with other aortic root dimensions [11]. The study also showed that Japanese population is also at higher risk of coronary artery occlusion owing to their smaller sinus of Valsalva diameters and low coronary heights. However, our study showed aortic dimensions somewhat comparable to European population. Also, the mean BSA of our study is also similar to European population. Having said that, mean left and right coronary heights in our population are similar to Japanese population. So, it is difficult to anticipate the risk of various complications in our population on the basis of available data.

Insights from one Singaporean study showed slightly smaller minimum and maximum aortic annular dimensions in their population than our study population (20.9 ± 2.4 mm vs 21.30 ± 3.96; 25.6 ± 2.5 mm vs 27.59 ± 4.44 mm, respectively). Furthermore, the study showed favorable outcomes in patients having smaller aortic annulus similar to Western population [12].

There is very limited data available which compare patients who have undergone TAVI for the difference in their aortic annular sizes. Watanabe et al. for the first time compared Asian cohort with European cohort for their clinical outcomes after TAVI. Both populations showed significant differences in their physique and aortic valve area. Despite these differences, the rate of intraprocedural complications and post-procedural outcomes were similar between two cohorts [13].

K F Kong et al. showed from his echocardiography based data that Asian population with bicuspid valve has significantly larger indexed aortic annulus diameters, along with diameters of sinus of Valsalva and sinotubular junction as compared to the European population [13]. This might be the reason that overall size of aortic annulus in our population appears similar to European population, as 35% patients in our study have bicuspid valve. However, when analyzing separately, the mean difference in aortic root dimensions between tricuspid and bicuspid aortic valve in our population is not significant.

There is diverse interracial and ethnicity difference between different Asian populations. Most of the literature available for Asian population comes from Japanese population [14–16]. Various studies conducted did not show any difference in outcomes when comparing them to the population having larger physique. Yet these studies give valuable perspectives, they may not fully elucidate the diversity present in various Asian subpopulation. Particularly, our study population showed body surface area larger than Japanese population. So, separate outcome studies are needed in our population.

### Limitations

This a single-center study so, the generalizability of results is not possible. Furthermore, it is a retrospective observational study so biases associated with retrospective nature of the study cannot be ruled out. Also, we did not take account of ilio-femoral sizes and data have shown that vascular complications are associated with increased mortality [17].

## Conclusion

Our study provides valuable insights into the anatomical characteristics of patients undergoing TAVI, particularly in an Asian cohort. The findings, including comparable aortic annular dimensions to European populations and the prevalence of bicuspid aortic valve morphology, contribute to the understanding of TAVI outcomes in this demographic. The limited representation of Asian populations in TAVI trials underscores the need for further research tailored to these patients, particularly regarding valve sizing and procedural safety. Our results emphasize the importance of considering anatomical differences in the development and selection of transcatheter heart valves to enhance patient outcomes in diverse populations.

## Data Availability

The datasets used and/or analyzed during the current study are available from the corresponding author upon reasonable request.

## List of Abbreviations

BAV: Bicuspid valve
BMI: Basal metabolic index
BSA: Body surface area
CT: Computed tomography
EF: Ejection fraction
LCC: Left coronary cusp
LVOT: Left ventricular outflow track
RCC: Right coronary cusp
SOV: Sinus of Valsalva
STJ: Sinotubular junction
TAVI: Transcatheter aortic valve implantation
TEE: Transesophageal echocardiography
